# New and Emerging Strategies in Platelet-Rich Plasma Application in Musculoskeletal Regenerative Procedures: General Overview on Still Open Questions and Outlook

**DOI:** 10.1155/2015/846045

**Published:** 2015-05-05

**Authors:** Francesca Salamanna, Francesca Veronesi, Melania Maglio, Elena Della Bella, Maria Sartori, Milena Fini

**Affiliations:** ^1^Laboratory of Biocompatibility, Innovative Technologies and Advanced Therapies, Department Rizzoli RIT, Rizzoli Orthopedic Institute, Via di Barbiano 1/10, 40136 Bologna, Italy; ^2^Laboratory of Preclinical and Surgical Studies, Rizzoli Orthopedic Institute, Via di Barbiano 1/10, 40136 Bologna, Italy; ^3^Department of Specialized, Experimental, and Diagnostic Medicine, University of Bologna, Via Massarenti 9, 40138 Bologna, Italy

## Abstract

Despite its pervasive use, the clinical efficacy of platelet-rich plasma (PRP) therapy and the different mechanisms of action have yet to be established. This overview of the literature is focused on the role of PRP in bone, tendon, cartilage, and ligament tissue regeneration considering basic science literature deriving from *in vitro* and *in vivo* studies. Although this work provides evidence that numerous preclinical studies published within the last 10 years showed promising results concerning the application of PRP, many key questions remain unanswered and controversial results have arisen. Additional preclinical studies are needed to define the dosing, timing, and frequency of PRP injections, different techniques for delivery and location of delivery, optimal physiologic conditions for injections, and the concomitant use of recombinant proteins, cytokines, additional growth factors, biological scaffolds, and stems cells to develop optimal treatment protocols that can effectively treat various musculoskeletal conditions.

## 1. Introduction

The vulnerability of the musculoskeletal system to acute or chronic injuries is often dramatic and, according to the WHO, they are the most common causes of severe long-term pain and physical disability affecting hundreds of millions of people worldwide [1]. Thus, bone, cartilage, tendon, and ligament injuries have serious socioeconomic consequences; for example, osteoarthritis affects nearly 27 million Americans or 12.1% of the adult population of the United States with a total annual cost of about $89.1 billion [2]. Besides osteoarthritis, also bone fracture care in osteoporotic patients has a high incidence with an annual cost of about $17 billion [3]. Similarly, ligamentous and tendinous injuries are very common with an annual incidence estimated at about 1 per 1000 people [4, 5]. However, the bulk of these musculoskeletal injuries does not heal with conservative managements and frequently requires surgery with several hardships for the patients. One of the most innovative methods used to biologically enhance tissue healing and regeneration includes the use of autologous blood products and, in particular, platelet-rich plasma (PRP). Blood is withdrawn from a patient's peripheral vein and centrifuged to achieve a high concentration of platelets (PLTs) within a small volume of plasma. It is reinjected at a site of injury or inserted as a gel or in combination with other biomaterials during surgery. At baseline levels, PLTs function as a natural reservoir for growth factors (GFs) and plays an important role in tissue healing and regeneration. GFs secreted by PLTs include platelet-derived growth factor (PDGF), epidermal growth factor (EGF), insulin-like growth factor (IGF-I), transforming growth factor *β*-I (TGF*β*-I), vascular endothelial growth factor (VEGF), hepatocyte growth factor (HGF), and basic fibroblast growth factor (bFGF), which provide the potential to modulate the healing of many tissues through interaction with specific cells [6, 7] (Table 1). This wide variety of GFs contributes to multifaceted roles of PRP, including the enhancement of anabolism, bone and vessel remodeling, cell proliferation, angiogenesis, inflammation control, coagulation, and cell differentiation [8].

Despite the lack of high-quality clinical trial data, several studies confirmed PRP clinical efficacy in the treatment of different types of musculoskeletal injuries [132–139]. However, many important questions remain unanswered. To reach a consensus on PRP use, there is the need of explaining why the employment of PRP generates different clinical results. The main drawback in evaluating the clinical effects of PRP is the inconsistency in established preparation protocols. To date, more than 40 commercial systems exist which claim to concentrate whole blood into a PLT-rich substance but a standardized preparation system has yet to be implemented in the common practice. Therefore, it is highly important for the clinician to be mindful of the different ways to obtain PRP and how the different methods affect the composition of PRP at the time of treatment [140]. The most important differences between the protocols and machines currently used are blood volume (from 9 to 120 mL), PRP volume (from 3 to 32 mL), activators (CaCl_2_, thrombin, batroxobin, bovine thrombin, and thrombin added to CaCl_2_), number of spins during centrifugation (1 or 2), and PLT concentration (from 1x to 18x) [141, 142]. Additionally, the presence or absence of leukocytes, which contain considerable amounts of VEGF could further affect the quality of PRP and consequently its effects [143–145]. In fact, a recent study by Kaux et al. demonstrated that a local infiltration of PRP, without both erythrocytes and leukocytes and obtained with the apheresis system, associated with submaximal eccentric protocol can improve symptoms of chronic jumper's knee [146]. Finally, the quality of PRP and resulting effects could also be influenced by patient's age, gender, body mass index, comorbidities, ethnic origin, healing capabilities, and different lifestyles (smoke, alcohol abuse, obesity, etc…) [147, 148].

The huge literature about this topic, from basic science reviews to* in vitro* and* in vivo* research, as well as clinical studies, highlighted the need of validated classification systems to compare the crucial differences between PRP preparation protocols. Among those proposed, we considered the PAW classification which assigns a code based on PLT concentration (PLTs/*µ*L), kind of activation (endogenous/exogenous), and white blood cell concentration (total WBCs and neutrophils) [149]. This paper is planned to give an overview of the last decade on the* in vitro* and* in vivo* studies on PRP in musculoskeletal regeneration also evaluating the different preparation protocols. Bone, cartilage, tendon, and ligament regeneration was considered.

## 2. Search Strategies

To identify the studies to be considered in the current review, a PubMed database search was performed using the following MeSH: “platelet-rich plasma” and “regeneration”. The searching limits were English language and papers published from July 8, 2004, to July 8, 2014. Three authors (Francesca Salamanna, Francesca Veronesi, and Melania Maglio) evaluated all articles. Studies were included if they were available online,* in vitro* or* in vivo*, and regarding bone, cartilage, tendons, and ligaments, while they were excluded if title and abstract clearly refuted eligibility. Also reviews, letters, or comments to the editor and clinical studies were excluded. All the selections were performed independently in duplicate. Disagreement was resolved by consensus.

## 3. Results

### 3.1. Search Strategies

**Figure 1:**
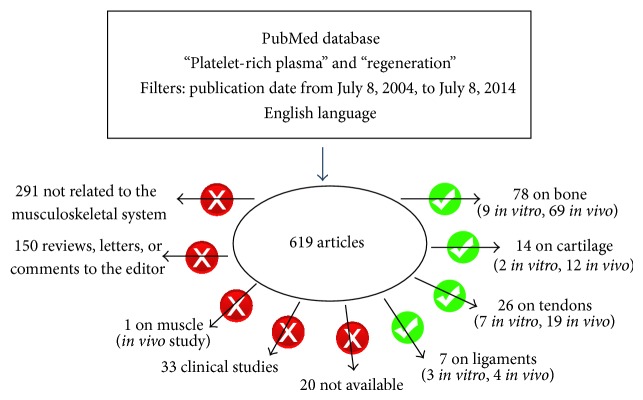
Schematic representation of the PubMed database searches.

**Figure 2:**
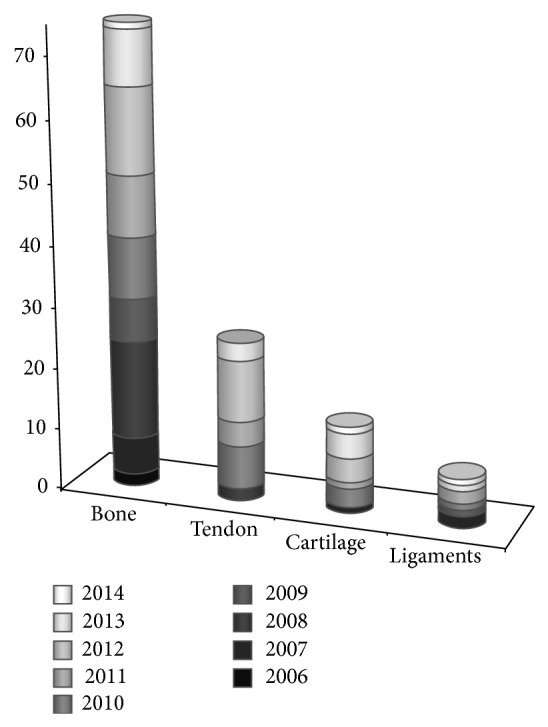
An overview on the application of PRP in musculoskeletal regenerative procedures in the last decade.

The PubMed search produced 619 articles. Several studies (494) were excluded: 290 were not related to musculoskeletal system, 150 were reviews, letters, or comments to the editor, 1 was on muscle regeneration, 33 were clinical studies on musculoskeletal system, and the other 20 were not available online to us. So a total of 125 articles were analyzed (Figure 1). In Figure 2, the number of papers for each tissue and year is reported (Figure 2).

Regarding bone tissue, the reviewed* in vitro* studies were carried out on osteoblasts (OBs) or mesenchymal stem cells (MSCs) with PRP combined or not with scaffolds.* In vivo* studies were performed with PRP alone or with autologous bone/scaffolds/cells or with a combination of scaffolds and cells. For tendon tissue regeneration, the examined* in vitro* studies evaluated the effects of PRP alone or with MSCs and scaffold on tenocytes or tendon tissue explants. In the* in vivo* studies, PRP was employed alone or associated with scaffolds, cells (mainly MSCs), or their combination. Concerning the* in vitro* studies on cartilage, PRP alone or with scaffold was evaluated on human chondrocytes, while, in* in vivo* ones, PRP was used in association with scaffolds or cells (chondrocytes or MSCs), also in combination with microfractures. As for anterior cruciate ligament (ACL) reconstruction, the* in vitro* studies evaluated the ACL fibroblast behavior under the effect of PRP with or without scaffolds while the* in vivo* evaluations were performed with PRP alone or in combination with scaffolds.

The main variables found among studies under review are presented in Table 2, while all the basic science literature derived from* in vitro* and* in vivo* studies were summarized in Tables 3 and 4.

### 3.2. PRP Biology: What Have We Learned?

Before examining PRP effects in musculoskeletal regeneration, a brief overview on its biology is provided below.

#### 3.2.1. Terminology and PLTs Products

Even though PRP is a generic term, many definitions and acronyms have appeared to differentiate PRP constituents and state of activation but may be also increasing the confusion. Although many authors urge standardization, the variety of names unfortunately does little to help standardize the product. PRP or PRF (platelet-rich fibrin) is the most used acronyms to indicate PLTs concentrates. Their processing techniques allow discarding the nonclinical useful elements, such as most of red blood cells, to concentrate the therapeutic effective ones, such as PLTs, GFs, leukocytes, or fibrinogen/fibrin. Actually, PRP products are divided into 4 families, based on leukocytes and fibrin content: pure platelet-rich plasma (P-PRP), leukocyte- and platelet-rich plasma (L-PRP), pure platelet-rich fibrin (P-PRF), and leukocyte- and platelet-rich fibrin (L-PRF) [150]. The first, also known as plasma rich in growth factors (PRGF), and the second are usually in the form of gel or liquid and are characterized by a low-density fibrin network, without or with leukocytes, respectively. On the other hand, the third, also named platelet-rich fibrin matrix (PRFM), and the fourth contain high-density fibrin network and exist only in the gel form. P-PRF is without leukocytes, while L-PRF contains leukocytes. It is clear that these four variables alone allow many possible variants of PRP to be produced; however, they provide a simple baseline for comparison.

#### 3.2.2. PLTs Number and *α*-Granule Contents

In healthy humans, the average PLT concentration of whole blood is around 200,000/*µ*L (normal range 150,000 to 350,000/*µ*L) [151]. PLTs are small anucleated cytoplasmic fragments of megakaryocytes normally thought as the responsible agents for hemostasis. Not only are the PLTs central to the clotting cascade, but they are also fundamental to tissue healing. The first step of the healing process is clot formation and PLTs activation [151]. Then biologically active molecules, GFs, and differentiation factors, are released from the *α*-granules [152, 153]. About 70% of the GFs are secreted within the first 10 minutes next to activation and, within the first hours, almost 100% have been secreted [154]. According on where they are in course of their life, several PLTs will die within a few days while some others may last up to 9 days ongoing to generate further GFs [155]. As previously mentioned, the degranulation of *α*-granules result in the release of a number of GFs, such as PDGF, EGF, IGF-I, TGF *β*-I, VEGF, HGF, and bFGF (Table 1). However, other bioactive factors, which include adhesive proteins, clotting and fibrinolytic factors and their inhibitors, proteases and antiproteases, antimicrobial proteins, and membrane glycoproteins, are getting increased attention in the last decade [153]. Another aspect is that *α*-granules also contain monocytes mediators and different interleukins (ILs) and chemokines, such as IL-1 *β*, IL-8, and MIP-1-2-3, regulated on activation, normal T cells expressed and secreted (CCL5), and more others, which are capable of mediating inflammation, stimulate cells chemotaxis, proliferation, and maturation [153, 156, 157].

Although PLTs have now been shown to store and release such a wide range of biologically active proteins, different enigmas, regarding their contents and possible activities on tissue healing, still remain to be solved.

#### 3.2.3. Methods of PRP Activation

Different methods of activating PRP influence the concentration of GFs. PRPs are frequently activated by calcium chloride, thrombin, chitosan, and batroxobin. Calcium chloride and thrombin activation are the two most common methods; 5% calcium chloride treatment for 19 min produces the most effective PRP, which has properties for soft-tissue adhesion [158]. Chitosan can be used instead of thrombin because it enhances aggregation, adhesion, and expression of *α*-granule membrane glycoprotein. Some data, however, suggest that exogenous thrombin activation of PRP may actually diminish its ability to induce bone formation compared with nonthrombin-activated PRP [159].

#### 3.2.4. Inter- and Intraspecies Variability

Preclinical models offer fundamental basis for the development of clinical treatments, although it is necessary to consider inter- and intraspecies variability principally in terms of PLTs count. As reported by Mitruka and Rawnsley [160] each species has its own number of PLTs, also with a wide range within the same species. Thus, the knowledge of the exact number of PLTs, when an animal model is used, is fundamental for understanding the effectiveness of the PRP application. Additional variability is added by some species-specific peculiarities [148]. This important variability needs to be considered in evaluating results from different animal models because it could be one of the reasons for dissimilar results obtained when PRP is used, as also demonstrated by the studies described below.

#### 3.2.5. Safety Profile

It is well known that PRP derive from autologous blood and this implies minimal risks for disease transmission, immunogenic reactions, and cancer [161]. GFs act on cell membranes rather than on the cell nucleus and activate normal gene expression [161]; they are not mutagenic and act through gene regulation and normal wound healing feedback control mechanisms.

Considering the long-term clinical experience with the use of PRP in oral and maxillofacial field, its use is considered to be safe [162, 163]. Differently, no long-term studies with PRP exist in the musculoskeletal field, despite a large number of treated patients [164]. Recently, a nonrandomized, prospective, longitudinal study on 808 patients indicated no adverse effects following injection of PRGF into the knee joint at 6 months [165]. Contrary, a recent case report reported an exuberant inflammatory reaction after 1 injection of PRP to treat jumper's knee in a 35-year-old male type 1 diabetic patient, revealing that PRP should be proposed only after careful consideration in cases of patients with morbidity risks [146]. Although the adverse effects are unusual, as with any injection, there is always a slight risk of injection site morbidity, infection, or injury to nerves or blood vessels. Scar tissue formation and calcification at the injection site are remote risks [166]. Infrequently, development of antibodies against clotting factors V and IX leading to life threatening coagulopathies has been reported [161, 167]. To date, no convincing preclinical studies and clinical trials demonstrating systemic effects following local PRP injections are reported and, as showed by Dhillon et al., this is probably due to the limited need of PRP injections in clinics and the short* in vivo* half-lives and local bioavailability of GFs produced by PRP [168].

## 4. The Role of PRP in the Regeneration of Bone

### 4.1. *In Vitro* Studies

Several studies [9–13] evaluated the* in vitro* effect of PRP showing that it was able to induce proliferation and osteogenic activity of human OB and OB-like cells. Additionally, Parsons et al. [14] investigated the effect of PRP on the osteogenic potential of human MSCs, suggesting the promotion of OB differentiation.

Bukharova et al. [15] developed a construct using a highly purified bone matrix as scaffold and osteogenic committed human adipose derived stem cells (ADSCs) together with PRP, later activated with thrombin/calcium chloride (CaCl_2_). While giving no real clues on the effect of PRP, the paper showed the creation of a construct that may be suitable for bone tissue engineering. Finally, Simson et al. [16] detected that the combination of an injectable chondroitin sulfate tissue adhesive and PRP with human MSCs could support bone growth.

More recently, Perut et al. [17] investigated the efficacy of different components of PLT concentrates on the osteogenic differentiation of BMSCs. Comparing two different procurement techniques, the authors reported that, in addition to the differences in PLT recovery between systems, the composition of PRP was associated with variance in the progressive release of bFGF from the platelet gel, which is associated with the proliferation of BMSCs and their ability to mineralize. The authors concluded that the ability of different PLT gels to induce proliferation and osteogenic differentiation of BMSCs was related to the composition of PRP including the platelet, leukocyte, and GF concentrations and availability.

*At a Glance*. (1) PRP addition in culture medium of MSCs, both BMSCs and ADSCs, and OBs improved proliferation and osteogenic activity; (2) the ability of different PLT gels to induce proliferation and osteogenic differentiation of BMSCs was related to the PRP composition (Table 3).

### 4.2. *In Vivo* Studies

Clots of PRP, PLT-rich GF (PRGF), and PLT-rich fibrin (PRF) were studied in different experimental conditions (sheep sternal wounds, critical size defect in rat calvaria, tibia and femurs, and nude mice calvaria bone defect) with good results in terms of bone regeneration [30–35] and promotion of the expression of TGF-*β* and bone morphogenetic protein-2 (BMP-2) [34]. Additionally, Messora et al. [33] observed a better outcome for PRP activated by CaCl_2_ in comparison to PRP activated by thromboplastin. Contrary to the above mentioned studies, Torres et al. [36] showed no beneficial effect of PRP on osseous regeneration in rabbit calvaria. Regarding the effect of topical application of PRP and platelet-poor plasma (PPP), it was compared in a rabbit model of full thickness calvaria defects, noticing better results for PRP [37]. Two studies focused on the application of PRP in osteoporosis [38, 39]. Chen et al. [38] administered different concentrations of PRP to promote the healing in osteoporotic rat femur. The results highlighted that, if on the one hand PRP enhanced bone regeneration, on the other hand too high concentrations could prevent a complete healing. Interestingly, Liu et al. [39], instead, used PRP to demonstrate its ability to prevent and treat osteoporosis by controlling the ratio of osteoblast and adipocyte in ovariectomized female mice. The study detected that PRP treatment improved bone quality in osteoporotic mice via promoting osteogenesis while suppressing adipogenesis in the bone marrow.

PRP was also added to autografts [40–45], Bio-Oss [46, 47], and fresh frozen bone allograft [48] in different animal models (i.e., critical size defects in mini. pigs, rat calvaria, and rabbit femur and tibia) and improved bone regeneration. In addition, Nagata et al. [44] explored the influence that the different proportion between particulate autogenous bone grafts and PRP (50, 100, 150 *µ*L) could exert on rat calvaria healing. The dose of 100 *µ*L of PRP proved to be the most effective in promoting bone formation, while the inhibitory effect of the highest PRP doses was noticed. However, other authors, using various animal models, found no benefits when PRP was added to autologous bone [49–53], autologous bone and Bio-Oss [55, 56], and xenografts [57].

Besides the use of PRP in combination with autografts, allografts, or xenografts, numerous studies have focused their attention on the PRP association with other synthetic and biologic materials such as ceramics (hydroxyapatite, HA, bioglass, calcium phosphate, CaP, and beta-tricalcium phosphate (*β*-TCP)) [58–65], metals [66], polymers (polyglycolic acid (PGA)) [67], composites (polycaprolactone-20% tricalcium phosphate (PCL-TCP)) [68], hydrogels [69, 70], alginate [71], coral [72, 73], and chitosan [74]. The majority of studies obtained good outcomes regarding the bone regenerative potential when PRP was added to the above mentioned materials [58–74]. Additionally, a significant bone formation was observed when PRP was used with biphasic CaP or PGA containing BMP-2 [64, 67]. A different application was proposed by Paulo et al., which treated rabbit fibula fracture with PRP and daily hyperbaric oxygen therapy sessions, with promising results [75]. However, several studies found opposite outcomes [76] compared to those just quoted, in particular, when PRP was used in association with ceramic [77–80], metallic [81], or composite materials [82]. A work by Clafshenkel et al. [80], exploring the association of melatonin-calcium aluminate scaffold with the addition of PRP in an ovariectomized rat model of calvaria defect, explained the failure in promoting bone regeneration with a possible conflict between the proliferative thrust induced by PRP and the differentiative stimuli mediated by melatonin.

Newly formed bone in rabbit [83, 84] and mice [85] calvaria defects were also obtained using PRP and bone marrow mesenchymal stem cells (BMSCs). Niemeyer et al. [86], also using a large animal model, observed that the presence of PRP could in part balance the differences in osteogenic potential of BMSCs and ADSCs. Kawasumi et al. [13], instead, evaluating PRP with increasing concentration of PLTs in combination with BMSCs in rat limb-lengthening model, detected a better qualitative regeneration of bone tissue using the higher PRP concentration. Lastly, Liu et al. [87], also in a study on heterotopic site of nude mice, testing a novel injectable tissue-engineered bone combined with induced hADSCs resuspended in PRP, showed an improvement in bone formation.

Comparing the contribution of MSCs and PRP to the regenerative capacity of ceramic bone substitutes, several studies indicated that the combined use of the three elements got better results in terms of osteogenesis [88–91]. Additionally, Kasten et al. [89] showed that over the positive effect of PRP with MSC and ceramic material on bone healing, an effect on vascularization was also proven. Batista et al. [92], instead, proved the effectiveness in the repair of rabbit tibial defects of PRP compared to bone marrow concentrate added separately to *β*-TCP scaffold, while Zhong et al. [93] obtained comparable results between PRP and bone marrow aspirate concentrate in combination with *β*-TCP in nude mice. Additionally, Behnia et al. [94] combined PRGF with a scaffold designed as carrier for GFs and stem cells, proving not only the applicability of the material but also the good potentiality in promoting bone regeneration when combined with PRGF and MSC. Man et al. [12] also tested the angiogenic and osteogenic potential of alginate microspheres combined with ADSCs and increasing percentage of PRP, demonstrating a high rate of mineralization in a model of nude mice with the presence of new vessel formation, with 10 and 15% of PRP. Finally, Zhang et al. [95] evaluated the immunogenicity of allogeneic PRP and the effect of a construct of allogeneic PRP/deproteinized bone matrix/autologous MSCs, with promising results not only regarding immunity but also for bone healing and vascularization. Contrary to the above mentioned study, Khojasteh et al. [169] evaluated the different contribution of PRP and BMSCs to various materials in rat calvaria defect, observing better bone formation with BMSCs alone as compared to their combination with PRP.

*At a Glance*. (1) Clots of PRP, PRGF, and PRF improved bone regeneration, promoting expression of TGF-*β* and BMP-2; (2) topical application of PRP showed better results in comparison to PPP; (3) PRP in osteoporotic animal models promoted bone healing; (4) PRP addition to autografts, Bio-Oss, fresh frozen bone allografts, or other synthetic and biologic materials showed discordant results in term of bone healing; (5) PRP in association with BMSCs or ADSCs, also in combination with different materials, showed good bone regeneration (Table 4).

## 5. The Role of PRP in the Regeneration of Tendons

### 5.1. *In Vitro* Studies

Several* in vitro* studies observed good results with different PRP formulations or PRP associated with scaffolds and BMSCs on tenocytes or tendon culture explants. It was observed that the addition of PRP to the culture medium counteracted the inhibition of tenocytes viability and proliferation induced by the osteoblasts-tenocytes coculture system [18] or by ciprofloxacin or dexamethasone [19]. In addition, some studies compared different PRP formulations. Platelet-poor clot releasate (PPCR) or leukocyte-reduced PRP (lrPRP) showed better results than platelet-rich clot releasate (PRCR) or high-concentration PRP (hcPRP), respectively. Indeed PPCR or lrPRP increased DNA content and total collagen and decreased VEGF-A, TGF-*β*1, metalloproteinases (MMP) expression [20], and proinflammatory cytokines in tenocytes or flexor digitorum superficialis tendon explants [21].

It was also observed that the best results were found in tenocytes with PRP gel activated with calcium and thrombin (PRP-Ca-Thr) in comparison to that activated with calcium (PRP-Ca) [22] and after the addition of PRFMembrane eluent in comparison to PRFMatrix ones in tenocytes medium [23].

The addition of PRP to collagen patch seeded with BMSCs improved biomechanical and histological features of digitorum profundus tendon in* in vitro* repair model [24].

*At a Glance*. PRP added to the culture medium of tenocytes or tendon explants improved viability and proliferation (Table 3).

### 5.2. *In Vivo* Studies

The effects of PRP alone were evaluated in acute lesions of rat supraspinatus, horse superficial digital flexor, rat rotator cuff, rat patellar, rabbit intrasynovial flexor, and sheep and rat Achilles tendons. An improvement in biomechanical, collagen fiber orientation, metabolic activity properties, and extracellular matrix (ECM) gene expression and a decrease in inflammatory cell number, vascularity, IGF-1, and TGF-*β* were observed [96–105]. In addition, platelet-rich growth factor (PRGF) and PRP with fibrin matrix (PRPF) showed the same results [106, 107]. On the contrary, no significant improvements were observed after the injection of PLT concentration (PC) in patellar tendons [108].

Also the combinations of PRP formulations with cells or scaffolds were studied. No synergic effect on sheep digital flexor healing was shown, when PRP was combined with peripheral blood MSCs (PBMSCs) [109]. On the other hand, the best results were observed when PRP was combined with ADSCs [110] or platelet-rich plasma fibrin matrix (PRPFM) with cross-linked acellular porcine dermal patch (APD), respectively, in rabbit and sheep Achilles tendon lesions [111, 130].

The use of PRP, collagen sponge, and tendon stem cells (TSCs) improved histological parameters and Coll I and Coll III expressions and productions of rat Achilles tendon lesions, especially after physical activity [112].

Finally, after the injection into mice abdominal cavities, tenocytes precultured with platelet-rich plasma-clot release (PRCR) induced high collagen production and tenocyte markers expression [113].

*At a Glance*. (1) On tendon lesions, PRP improved biomechanical, collagen fibers orientation, metabolic activity properties, and ECM gene expression with a decrease of inflammatory cell number, vascularity, IGF-1, and TGF-*β*; (2) PRP and PBMSCs combination did not improved tendon healing; (3) PRP combined with ADSCs or PRPFM with cross-linked APD improved tendon healing (Table 4).

## 6. The Role of PRP in the Regeneration of Cartilage

### 6.1. *In Vitro* Studies

PLT-derived GFs are proteins with the capacity to stimulate chondrocytes to regenerate cartilage. PRGF-treated chondrocytes showed markedly increased synthesis of proteoglycans and collagen. Plasma rich in GFs is an excellent vehicle for GFs, especially PDGF and TGF-*β*. In fact, several studies have documented the effectiveness of GFs in chondrogenesis and prevention of joint degeneration by controlling the synthesis and degradation of extracellular matrix proteins. Their mode of action is to bind to the extracellular domain of a target GF receptor, which in turn activates the intracellular signal transduction pathways.

Wu et al. [25] evaluate the effect of collagen matrix on the regeneration potentials of PRP for chondrocytes homeostasis showing that collagen matrix stimulated integrins and CD44 signaling was coordinated with the addition of PRP. These interactions play a critical role in regulating cell proliferation, chondrogenic and inflammatory gene expressions, and matrix remodeling of human articular chondrocytes. The study demonstrated a schematic model of collagen matrix cooperating with PRP to inhibit the ECM degradation and promote ECM synthesis and deposition. Recently, Cavallo et al. [26] assessed the effect of various PRP formulations on human chondrocytes. Results showed that PRP with a relatively low concentration of platelets and very few leukocytes stimulated chondrocyte appearing to favor some mechanisms that stimulate chondrocyte anabolism, as demonstrated by the expression of type-II collagen and aggrecan, whereas PRP with high concentrations of both platelets and leukocytes appeared to promote other biological pathways involving various cytokines. This might be due to the presence of leukocytes in PRP; the leukocytes may have been responsible for the increased expression of certain molecules such as IL-1b, IL-6, VEGF, and FGF-b, which in turn could have stimulated TIMP-1 and IL-10.

*At a Glance*. (1) Collagen matrix and PRP promoted cartilage ECM synthesis; (2) PRP with a relatively low concentration of PLTs and very few leukocytes stimulated chondrocyte anabolism; (3) PRP with high concentrations of both PLTs and leukocytes appeared to promote chondrocyte catabolism (Table 3).

### 6.2. *In Vivo* Studies

Some* in vivo* studies evaluated the effects of PRP when combined with scaffolds (polymers, collagen, and demineralized bone matrix), cells (chondrocytes and MSCs), or a combination of them.

In rabbit chondral defects, PRP incorporated in poly(lactic-co-glycolic acid) (PLGA) successfully improved the healing [114], while, in sheep and goat osteochondral defects, PRP with collagen-HA scaffolds or demineralized bone matrix did not improve or even decreased the healing [115, 116]. Kon et al. showed not only the lack of a positive effect but also a negative influence of autologous PRP on bone and cartilage regeneration with amorphous cartilaginous repair tissue and a poorly spatially organized underlying bone tissue [115].

After the assessment of feasibility of PRP as injectable scaffold [117], an improvement in the repair of rabbit osteochondral defects after the implantation of PRP seeded with chondrocytes and a chondrocyte differentiation of BMSCs and ADSCs seeded within the PRP scaffold was observed [118]. Similarly, Lee et al. [119], using PRP gel embedded with synovial membrane derived mesenchymal stem cell (SDSCs), showed a substantial improvement in the repair of osteochondral defects in a rabbit model.

The combination of hydrogel scaffold, chondrocytes, and PRP promoted the* in vivo* healing of articular or nonarticular cartilage lesions, respectively, in rabbit and rat, revealing successful regeneration of hyaline chondrocytes with formation of perichondrium-like normal joint cartilage [120, 121]. On the contrary, the separate adding of PRP or BMSCs to already available composite biphasic scaffold, composed by PLGA, poly(glycolic acid), and calcium sulfate, resulted in a significantly better mini. pig osteochondral defect healing, but with no synergic effects [122]. To preserve the advantages of chondrocyte therapy in a single-stage approach to osteochondral defects, Marmotti and coworkers [123] offered a single-step therapeutic approach for osteochondral defects using autologous cartilage fragments loaded onto a scaffold composed of a hyaluronic acid derivative, human fibrin glue, and PRP, in a rabbit model. Finally, the same studies [124, 125] using sheep and rats, evaluated the effect of PRP combined with microfractures on healing of chondral defects, showing that PRP application in addition to microfractures resulted in a better cartilage healing than microfractures alone.

*At a Glance*. (1) PRP incorporated in PLGA improved cartilage healing; (2) PRP with collagen-HA or demineralized bone matrix did not improve or even decreased cartilage healing; (3) good quality results in cartilage regeneration when PRP was associated with chondrocytes or MSCs with or without scaffolds (Table 4).

## 7. The Role of PRP in the Regeneration of Anterior Cruciate Ligament

### 7.1. *In Vitro* Studies

Mastrangelo et al. [27] observed that porcine and ovine ACL fibroblasts within a collagen-platelet scaffold from skeletally immature animals have greater proliferation and migration potential than adolescent and adult cells. Similar results were obtained by Magarian et al. [28] observing the response to PRP treatment in human ACL fibroblasts derived from 5 skeletally immature and 5 adolescent patients. Yoshida et al. [29] evaluated the optimal concentration of PLTs (1x, 3x, and 5x) to stimulate ACL healing using porcine ACL fibroblasts, revealing that 1x PRP was the best stimulator while higher concentrations of PLTs had diminishing effects.

*At a Glance*. (1) ACL fibroblasts within a collagen-platelet scaffold from skeletally immature animals had greater proliferation than adolescent and adult cells; (2) 1x PRP was the best stimulator for ACL healing in ACL fibroblasts (Table 3).

### 7.2. *In Vivo* Studies

Several authors using different animal models, porcine and canine, demonstrated that healing of transected ACL could be enhanced with the use of a collagen-PRP hydrogel placed within the repair site [126–128] suggesting also that there was little functional difference in ligament healing with the use of collagen scaffolds saturated with 3x or 5x PRP [129]. Differently, Zhai and coworkers [130] showed that platelet-rich gel + deproteinized bone could trigger tendon-bone healing by promoting the maturation and ossification of the tendon-bone tissue in a rabbit model. Finally, the role of PRP in promoting revascularization and reinnervation during ACL healing was clarified, using a canine animal model [131].

*At a Glance*. (1) Healing of transected ACL enhanced with the use of a collagen-PRP hydrogel; (2) PLTt-rich gel + deproteinized bone triggered tendon-bone healing; (3) PRP promoted revascularization and reinnervation during ACL healing (Table 4).

## 8. Discussion

Bone, cartilage, tendon, and ligament injuries have serious socioeconomic consequences in terms of health, rehabilitation, and lost working hours. The rationale of the use of PRP is that it concentrates more PLTs than the whole blood, allowing the delivery of bioactive GFs and molecules that promote tissue healing. Recently, regenerative medicine and tissue engineering focused on the use of GFs [163] and cell-based therapy to improve the quality and speed of healing suggesting that this combined biological approach may be useful even for the treatment of recalcitrant overuse musculoskeletal injuries in highly demanding patients if the appropriate dose of cells and GFs is applied [170].

No of fewer importance, PLT-rich preparations may also improve long-term outcomes in patients expected to have impaired healing, such as those with harmful lifestyle choices (e.g., smoking), medications (e.g., steroids), comorbidities (e.g., diabetes, osteoporosis, atherosclerosis, and Alzheimer), and advanced age [171, 172].

The use of PRP is a quick, minimally invasive, and relatively low-cost therapeutic strategy and, for these reasons, from the last three decades, PRP injections have been studied as a therapeutic alternative for different musculoskeletal injuries. The present study evaluated the last 10 years preclinical results on regenerative medicine and PRP in the musculoskeletal tissues in order to summarize the most important findings on both positive and negative data and to stimulate further preclinical and clinical research. Until 2006 PRP was preclinically investigated mainly for bone regeneration but in the last few years, the number of studies on the treatment of cartilage, ligaments, and especially tendon lesions is increasing. Even if the preclinical results did not report adverse effects, there was a wide variability among the results making it impossible to draw a standard protocol or indication for the so different musculoskeletal injuries. First and foremost, the generalized nature of the terminology may be a probable barrier to differentiate between various products and their respective protocols and it is possible that the different PRP preparation techniques, doses, and application modalities produce different results. The other main differences emerging regarded the number of centrifugations, the withdrawn blood volume, the obtained PRP final volume, the different PLT concentrations, the presence or absence of leukocytes in the final preparation, and, lastly, the use of an activator. The above listed factors are subjected to a great variability and in many papers are not specified in detail. The adoption of one of the proposed classification systems (PAW classification), in order to compare data, was not always and completely applicable, making it impossible to reach a conclusion on the best PLT concentration to be used.

The application of PRP* in vitro* showed promising results in all examined tissues. Researchers on bone demonstrated that the addition of PRP in cell culture medium determined good proliferation and osteogenic activity of MSCs (both BMSCs and ADSCs) and OBs. The presence of PRP had a positive effect in the culture of tenocytes or tendon explants and promising results were also observed with chondrocytes and ACL fibroblasts. The* in vivo* protocols are even more varying and complex than the* in vitro* ones. In bone, a wide spectrum of defects in different anatomic locations have been analyzed (calvarium, radius, tibia, condyle, iliac crest, ulna, femur, fibula, sternum, spine, frontal bone, and skull), besides vessel and bone formations in ectopic sites, employing the combination of PRP with scaffolds or autologous bone. Despite some contrasting data,* in vivo* studies showed encouraging results when PRP was used, also in combination with MSCs with or without other cells.

Different PRP formulations have been used for the regeneration of the most important tendons of the body: patellar, Achilles, superficial or deep digital flexor, rotator cuff, and intrasynovial flexor tendons. Similar to the bone tissue, for* in vivo* tendon regeneration, good results were observed when PRP was employed.

Finally, contrasting findings were observed in partial thickness, full thickness, osteochondral defects, and ACL reconstruction, although the* in vivo* studies on cartilage regeneration reported good quality results when PRP was associated with chondrocytes or MSC with or without scaffolds. Regarding ACL, all examined* in vivo* studies showed high-quality results in terms of regeneration.

To summarize,* in vitro* studies underlined the role of PRP for tissue regeneration and, when comparing different PRP formulations, concluded that a specific range of PLT number is required in order to obtain the best results with an increase in ECM protein expression and a decrease in the levels of proinflammatory cytokines and MMPs,* via* downregulation of known catabolic signaling pathways. However, the* in vitro* positive effects were not confirmed in all the* in vivo* studies because of the many variables affecting the success rate in a complex scenario where both PRP and the lesion site play a crucial role.

## 9. Conclusions and Outlook for Future Research

Despite the fact that many of the examined studies showed the potential positive effect of PRP in the treatment of musculoskeletal diseases, there is a paucity of human randomized controlled trials to provide level I evidence for the efficacy of this intervention. In fact, most of the human studies are case series or retrospective studies without a control group. Generally, they are small in size and unpowered. Thus, further evaluations are recommended and future studies should (1) find uniform and standardized nomenclature and preparation protocols; (2) optimize the number of PLTs and leukocytes cells; (3) make a direct comparison with other therapeutic techniques; (4) increase the quality of preclinical trials on safety, efficacy, and proof of concept studies; (5) clarify the role of the patient and lesion characteristics together with the local inflammatory microenvironment in the clinical outcome.

## Figures and Tables

**Table 1:** Main GFs release by *α*-granules.

GFs	Mechanism of action

TGF-*β*	(i) MSC proliferation and differentiation (ii) Cell mitogenesis (iii) Collagen II, proteoglycan, and ECM synthesis (iv) Endothelial chemotaxis and angiogenesis (v) Macrophages and lymphocyte proliferation inhibition; chondrocyte differentiation (vi) TIMP upregulation

PDGF-a and -b	(i) OBs and MSCs mitogenesis (ii) Macrophages, neutrophil, and other cell chemotaxis; collagen I synthesis

bFGF	(i) Chondrocyte and OB differentiation (ii) MSCs, chondrocyte, and OB mitogenesis

EGF	(i) Endothelial chemotaxis and angiogenesis (ii) Collagen synthesis (iii) MSC and epithelial cell mitogenesis

CTGF	(i) Angiogenesis (ii) Cartilage regeneration (iii) Fibrosis (iv) Platelet adhesion

VEGF	(i) Angiogenesis (ii) Endothelial cell mitogenesis

IGF	(i) Cell proliferation (ii) Collagen synthesis (iii) Myoblast proliferation and differentiation

**Table 2:** Main variables of the reviewed studies and factors implicated in PRP efficacy.

Tissue type	Study type	Blood volume (mL)	PRP volume (mL)	PLT count (×10^6^/*μ*L PRP)	Leukocyte count (×10^4^/*μ*L)	Activators
Bone	*In vitro *	51 ± 30 (*n* = 4)	5.3 ± 6.6 (*n* = 2)	4.2 ± 6.6 (*n* = 4)	NS	Thrombin, CaCl_2_, Ca-gluconate (*n* = 4)
	*In vivo *	77 ± 135 (*n* = 54)	2.6 ± 4.9 (*n* = 34)	2.3 ± 2.3 (*n* = 34)	1.4 ± 4.1/*μ*L (*n* = 3)	Thrombin, CaCl_2_, CaCl_2_ + thromboplastin, Ca-gluconate, and CaCl_2_ + thrombin (*n* = 52)

Tendon	*In vitro *	93 ± 167 (*n* = 8)	4.5 ± 3.8 (*n* = 6)	1.9 ± 2.4 (*n* = 10)	4 ± 4.1/*μ*L (*n* = 2)	Thrombin, CaCl_2_, Ca-gluconate + thrombin (*n* = 5)
	*In vivo *	17 ± 16 (*n* = 15)	2.4 ± 1.2 (*n* = 9)	1.9 ± 1.6 (*n* = 9)	2 ± 3/*μ*L (*n* = 2)	Thrombin, CaCl_2_ (*n* = 6)

Cartilage	*In vitro *	115 ± 61 (*n* = 3)	1.0 ± n/a (*n* = 1)	0.9 ± n/a (*n* = 1)	NS	Thrombin, CaCl_2_
	*In vivo *	25 ± 20 (*n* = 11)	3.1 ± 3.8 (*n* = 8)	2.8 ± 3.9 (*n* = 8)	NS	Thrombin, CaCl_2_, Ca, Fibrinogen Thrombin (*n* = 4)

Anterior cruciate ligament	*In vitro *	33 ± 38 (*n* = 2)	NS	0.5 ± 0.2 (*n* = 3)	NS	NS
	*In vivo *	33 ± 23 (*n* = 3)	5.0 ± 5.7 (*n* = 2)	1.6 ± 0.7 (*n* = 5)	NS	Thrombin, CaCl_2_ (*n* = 1)

*n*: number of data available for the specific variable in the considered papers; NS: not specified.

**Table 3:** *In vitro* studies on musculoskeletal tissue regeneration. The PAW classification has been attributed when possible.

Cell type	PRP formulation	PRP combination	Blood volume (mL)	Centrifugation (number of spins)	PRP volume (mL)	Platelet concentration	Activator (+/−)	Leukocytes (+/−)	PAW classification	Reference
Alveolar bone hOBs	PRP	PRP	8.5 mL	2	0.6 mL	NS	NS	NS	Not applicable	[9]
Jaw bone hOBs	PRP	PRP	NS	NS	NS	10^6^/mL	Thrombin/Ca-gluconate	NS	P1-x	[10]
Purchased hOB-like cells	PRP	PRP	60 mL	2	NS	0.5–1.0 × 10^6^/*μ*L	2% alginate-6% CaCl_2_	NS	P2-x	[11]
Rabbits ADSCs	PRP	5–10–15% PRP + -microspheres	30 mL	2	NS	NS	500 U bovine thrombin in 1 mL 10% CaCl_2_	NS	x	[12]
Rat BMSC	PRP	L-M-H PRP + BMSCs	40 mL	2	NS	High: 4358 ± 265 × 10^3^ Medium: 1453 ± 88 × 10^3^ Low: 48 ± 29 × 10^3^ (platelets/*μ*L)	CaCl_2_/thrombin	NS	P4-x P4-x P1-x	[13]
Purchased hMSCs	PRP	PRP	80 mL	Caption device (no centrifuge)	10 mL	NS	Thrombin/CaCl_2_	+	x	[14]
Anterior abdominal wall hADSCs	PRP	PRP + OsteoMatrix	NS	2	0.5 mL/cm^2^	14 × 10^6^/mL	Thrombin/CaCl_2_	NS	P1-x	[15]
Purchased porcine MSCs	PRP	PRP + CS-NHS	NS	2	NS	NS	NS	NS	Not applicable	[16]
hBMSCs	PRP	P-PRP L-PRP	45 mL 150 mL	1 2	5 mL NS	260 ± 37 (83–738) 194 × 10^3^/*μ*L 920 ± 491 (555–1114) 912 × 10^3^/*μ*L	10% CaCl_2_	0.00 6.4 ± 6.5 (1.8–14.4) 5.7 × 10^3^/*μ*L		[17]
Purchased hOBs and tenocytes from semitendinosus and gracilis tendons	PRP	OBs and tenocytes cocultures + PRP	6 mL	2	1 mL	1.005 × 10^12^/L	NS	NS	P3	[18]
Human tenocytes from hamstring	PRP	Ciprofloxacin + dexamethasone + 10% PRP	55 mL	1	NS	353000–837000/*μ*L	Bovine thrombin	NS	P2-x	[19]
Human tenocytes from hamstring	-PRCR -PPCR	10%, 20% PPCR or 10%, 20% PRCR	500 mL	4	NS	PPCR: 6–11 × 10^6^/mL; PRCR: 7–19 × 10^6^/mL	CaCl_2_	PPCR and PRCR: 0.10 × 10^6^/mL	P1-x-B	[20]
Horse flexor digitorum superficialis tendon	-PRP -hcPRP -lrPRP -clPRP	PRP, hcPRP, lrPRP, or clPRP	PRP, plasma, and hcPRP: 60 mL; lrPRP and clPRP: 90 mL	NS	PRP: 10 mL; hcPRP and clPRP: 5 mL; lrPRP: 8 mL	PRP: 6 × 10^5^/uL; hcPRP: 12 × 10^5^/uL; lrPRP: 5 × 10^5^/uL; clPRP: 6 × 10^5^/uL	NS	PRP: 8 × 10^3^/uL; hcPRP: 24 × 10^3^/uL; lrPRP: 1 × 10^3^/uL; clPRP: 28 × 10^3^/uL	P2-B P3-A P2-B P2-A	[21]
Human tenocytes from the rotator cuff	PRP	10% PPP or PRP-Ca, or PRP-Ca-Thr	NS	NS	NS	1400 × 10^3^/uL	calcium gluconate ± thrombin	NS	P4-x	[22]
Canine patellar tendon	-PRFMembrane -PRFMatrix -BC	PRFMatrix or PRFMembrane or BC eluent	PRFMatrix: 9 mL; PRFMembrane: 18 mL; BC: 4 mL	2	2 mL	NS	PRFMatrix and PRFMembrane: CaCl_2_	NS	x	[23]
Canine flexor digitorum profundus tendon	PRP	PRP + collagen Patch + MSCs	55 mL	2	1 mL	Mean: 1316 × 103/*μ*L	Bovine thrombin/CaCl_2_	NS	P4-x	[24]
Immortalized human articular chondrocytes	PRP	PRP + collagen matrix	NS	1	NS	NS	Bovine thrombin	NS	x	[25]
Human chondrocytes from osteoarthritic cartilage	P-PRP L-PRP PPP	P-PRP L-PRP PPP	45 mL 150 mL 150 mL	1 2 2	1 mL NS NS	Median number: PLT × mm^3^ = 929000 PLT × mm^3^ = 194000 PLT × mm^3^ = 6000	CaCl_2_ CaCl_2_ CaCl_2_	− + −	P3-x P2-x P1-x	[26]
Porcine and ovine ACL fibroblasts	PRP	Collagen-platelet scaffold	60 mL	1	NS	NS	NS	NS	Not applicable	[27]
Human ACL fibroblasts	PRP	PRP	6 mL	2	NS	3 × that of the whole blood	NS	NS	P1	[28]
Porcine ACL fibroblasts	PPP PRP	PPP 1x PRP 3x PRP 5x PRP	NS	2 2 2 2	NS	8 × 10^6^ PLTs/mL 129 × 10^6^ PLTs/mL 370 × 10^6^ PLTs/mL 615 × 10^6^ PLTs/mL	NS	+	P1 P1 P2 P2	[29]

Not specified (NS), mesenchymal stem cell (MSC), adipose tissue (AT), stromal vascular fraction of AT (SVF AT), bone marrow cells (BMC), low PRP (lPRP), medium PRP (mPRP), high PRP (hPRP), growth factors (GF), CS-NHS (chondroitin sulfate succinimidyl succinate), calcium (Ca), osteoblast (OB), platelet-rich clot releasate (PRCR), platelet-poor clot releasate (PPCR), high-concentration PRP (hcPRP), leukocyte-reduced PRP (lrPRP), concentrated-leukocytes PRP (clPRP), blood clot (BC), mesenchymal stem cells (MSCs), PRP activated with calcium (PRP-Ca), PRP activated with calcium and thrombin (PRP-Ca-Thr), calcium chloride (CaCl_2_), white blood cells (WBC), low PRP (P-PRP), high PRP (L-PRP), and platelet-poor plasma (PPP).

PAW classification: P1 (≤baseline), P2 (>baseline-750,000), P3 (>750,000–1,250,000), P4 (>1,250,000), x (exogenous activation), A (above baseline), and B (below baseline).

**Table 4:** *In vivo* studies on musculoskeletal tissue regeneration.

Tissue type	PRP formulation	PRP combination	Blood volume (mL)	Centrifugation (number of spins)	PRP volume (mL)	Platelet concentration	Activator (+/−)	Leukocytes (+/−)	PAW classification	Reference
Mice dorsum	PRP	5–10–15% PRP + ADSCs-microspheres	30 mL	2	NS	NS	500 U bovine thrombin in 1 mL 10% CaCl_2_	NS	x	[12]
Rat limb	PRP	L-M-H PRP + BMSCs	40 mL	2	NS	High: 4358 ± 265 × 10^3^ Medium: 1453 ± 88 × 10^3^ Low: 48 ± 29 × 10^3^ (platelets/*μ*L)	CaCl_2_/thrombin	NS	P4-x P4-x P1-x	[13]
Sheep sternum	PRGF	PRGF	40 mL	1	NS	NS	CaCl_2_	NS	x	[30]
Rat tibia	PRP	PRP	20 mL	2	10–15% of total	1 × 10^6^/*μ*L	CaCl_2_	NS	P3-x	[31]
Rat calvaria	PRP	PRP	3.15 mL	2	0.35 mL	1852.307 ± 1084.85 × 10^3^/*µ*L	CaCl_2_	NS	P4-x	[32]
Rat calvaria	PRP	PRP	3.15 mL	2	0.35 mL	2977.66 ± 1174.83 × 10^3^/ *µ*L	10% CaCl_2_ 25% thromboplastin	NS	P4-x	[33]
Rat femur	PRP	PRP	NS	2	NS	NS	CaCl_2_/ thrombin	NS	x	[34]
Mouse calvaria	PRF	PRF	NS	NS	NS	NS	NS	NS	Not applicable	[35]
Rabbit calvaria	PRP	PRP	10 mL	1	~0.9 mL	NS	CaCl_2_	NS	x	[36]
Rabbit calvaria	PRP PPP	PRP PPP	130 mL	2	NS	900,000/*µ*L	Thrombin/ CaCl_2_	NS	P3-x	[37]
Ovx. rat femur	PRP	-H-PRP; -M-PRP; -L-PRP; -PPP	NS	3	NS	H-PRP: 8.21 ± 0.4 × 10^9^/mL; M-PRP: 2.65 ± 0.2 × 10^9^/mL; L-PRP: 0.85 ± 0.16 × 10^9^/mL; PPP: 8 ± 0.5 × 10^6^/mL	Thrombin/ CaCl_2_	NS	P4-x P4-x P3-x P3-x	[38]
Ovx. mice femur	PRP	PRP	NS	NS	NS	NS	NS	NS	Not applicable	[39]
Mini. pig tibia	PRP	PRP + AB	120 mL	1	20 mL	NS	Thrombin/ CaCl_2_	NS	x	[40]
Rabbit tibia	PRP	PRP + AB	10 mL	2	NS	NS	CaCl_2_/ thrombin	NS	x	[41]
Rabbit tibia	PRP	PRP + AB	10 mL	1	0.5 mL	NS	CaCl_2_	NS	x	[42]
Rat calvaria	PRP	PRP 50–100–150 *µ*L + AB	3.15 mL	2	0.35 mL	2611.80 ± 313.34 × 10^3^/*µ*L	CaCl_2_	NS	P4-x	[43]
Rabbit calvaria	PRP	PRP + AB	40 mL	1	NS	NS	CaCl_2_	NS	x	[44]
Rat calvaria	PRP	PRP 50–100–150 *µ*L + AB	3.15 mL	2	0.35 mL	2,718.46 ± 359.65 × 10^3^ platelets/*µ*L.	CaCl_2_	NS	P4-x	[45]
Rabbit femur	PRP	PRP + Bioss Collagen	4 mL	NS	NS	NS	NS	NS	Not applicable	[46]
Dog tibia	PRP	PRP + Bio-Oss	20 mL	2	NS	1.380.000	CaCl_2_/ thrombin	NS	Not applicable	[47]
Rat calvaria	PRP	PRP + FFBA	3.15 mL	2	0.35 mL	2,628.8 ± 603.07 × 10^3^/*µ*L	CaCl_2_	NS	P4-x	[48]
Rabbit calvaria	PRP	PRP + particulated AB	38 mL	1	5 mL	1,7 × 10^6^ ± 680,200/*µ*L	Thrombin/ CaCl_2_	13.42 × 10^9^/L	P4-x	[49]
Rabbit calvaria	PRP	PRP + AB	15 mL	2	1 mL	2,4 × 10^6^ ± 1,5 × 10^6^/*μ*L	CaCl_2_	NS	P4-x	[50]
Rabbit calvaria	PRP	PRP + AB	15 mL	2	1 mL	2.564 × 10^6^ ± 1.621 × 10^6^/*μ*L	CaCl_2_	NS	P4-x	[51]
Rabbit calvaria	PRP	PRP^*^ + AB PRP° + AB	10 mL	1^*^ 2°	1 mL^*^ 0.5 mL°	NS	CaCl_2_	NS	x	[52]
Rabbit calvaria	PRP	hPRP + AB	10 mL	2	1 mL	2.414.720 ± 1547.862 platelets/mL	CaCl_2_/ thrombin	NS	P1-x	[53]
Rabbit calvaria	PRP	PRP + AB + receptor bed	10 mL	2	NS	1,2 × 10^6^ platelets/mm^3^	Thrombin/ CaCl_2_	NS	P3-x	[54]
Goat's frontal bone	PRP	PRP + AB	250 mL	NS	NS	800 × 10^9^/L	CaCl_2_/ bovine thrombin	NS	P3-x	[55]
Goat's frontal bone	PRP	PRP + AB + BioOss	250 cm^3^	4	NS	800–1000 × 10^9^/L	CaCl_2_/ bovine thrombin	NS	P3-x	[56]
Rabbit femur	PRGF	PRGF + DBBM	10 mL	1	1 mL	NS	CaCl_2_	NS	x	[57]
Rabbit radius	PRP	hPRP + HA	500 mL	1	NS	2,422 × 10^9^L	NS	NS	P4	[58]
Mini. pig's subcutis	PRP	PRP + Bovine HA or Phycogenic HA or Bioglass	250 mL	NS	NS	483.8 ± 97.2 × 10^3^/*µ*L	NS	24.8 ± 8.9 × 10^3^/*µ*L	P2-A	[59]
Rabbit condyle	PRP	PRP + CPC	NS	NS	NS	NS	NS	NS	Not applicable	[60]
Mini. pig tibia	PRP	PRP + Ca-P	120 mL	2	20 mL	NS	Thrombin/ CaCl_2_	NS	x	[61]
Rat cranium	PRP	PRP + HA or *β*-TCP	NS	NS	NS	NS	CaCl_2_/ thrombin	NS	x	[62]
Rabbit radius	PRP	PRP + BG	10 mL	1	0.8 mL	NS	CaCl_2_/ thrombin	NS	x	[63]
Rabbit calvaria	PRP	PRP + rhBMP2-BCP	10 mL	2	NS	78.1 ± 1.62 × 10^4^/mm^3^	CaCl_2_/ thrombin	NS	P3-x	[64]
Rabbit iliac crest	PRP	PRP + ringed PTFE vascular grafts + HA or collagen gel beads	5 mL	2	0.18 mL	1,100 ± 332 × 10^3^/*µ*L	NS	NS	P3	[65]
Rabbit radius	PRP	PRP + Ti + Bone	10 mL	2	1 mL	1.0– 1.5 × 10^12^ thrombocytes/L	Thrombin/ CaCl_2_	NS	P3-x	[66]
Nude rat calvaria	PRP	PRP + PGA + rhBMP-2	NS	NS	NS	NS	CaCl_2_/ thrombin	NS	x	[67]
Rat femur	PRP	PRP + PCL-TCP	10 mL	2	1.5 mL	~600 × 10^3^platelets/mL	CaCl_2_/ thrombin	NS	P2-x	[68]
Rat ulna	PRP	PRP + gelatin hydrogel + SEW2871-micelles	10 mL	2	1 mL	NS	CaCl_2_	NS	x	[69]
Rabbit calvaria	PRP	Activated/inactivated PRP + gelatine hydrogel	NS	NS	~0.8 mL	NS	Thrombin/ CaCl_2_	NS	x	[70]
Cattle hoof	PRP	PRP + Gelatin microspheres + alginate	NS	2	1 mL	152.8 ± 98.7 × 10^4^ platelets/*μ*L	CaCl_2_	NS	P4-x	[71]
Rabbit radius	PRP	hPRP + Persian Gulf Coral	500 mL	NS	NS	2422 × 10^9^/L	NS	NS	P4	[72]
Rabbit radius	PRP	hPRP + coral	NS	NS	NS	2422 × 10^9^/L	NS	NS	P4	[73]
Rabbit cranium	PRP	PRP + chitosan sponge	8 mL	2	0.6-0.7 mL	NS	CaCl_2_	NS	x	[74]
Rabbit fibula	PRP	APC + HBO	2.5 mL	1	1.5 mL	NS	NS	NS	Not applicable	[75]
Beagles calvaria	PRP	PRP + PDBM or Lactosorb or BMP	27 mL	1	3 mL	NS	CaCl_2_/ thrombin	NS	x	[76]
Goats cranium	PRP	rPRP or hPRP or gPRP + HA or *β*-TCP; hPRP + bone	500 mL^h^ 250 cm^3 g^	NS	NS	NS	CaCl_2_/ thrombin	NS	x	[77]
Rabbit calvaria	PRP	PRP + *β*-TCP	8 mL	2	0.7 mL	NS	NS	NS	Not applicable	[78]
Rabbit spinal fusion	PRP	PRP + Calcium Carbonate + HA	54 mL	2	10 mL	NS	thrombin/ CaCl_2_	NS	x	[79]
Ovx. rat calvaria	PRP	PRP + CA + Mel	4 mL	NS	325 *µ*L	NS	NS	NS	Not applicable	[80]
Porcine skull	PRP	PRP + ankyloss graft	NS	NS	NS	118 ± 12 thrombocytes 1000/*µ*L	CaCl_2_/ thrombin	4.3 ± 1.9 leukocytes 1000/*µ*L	P1-x-B	[81]
Goats tibia	PRP	PRP liquid or PRP gel + non-coated or Ca-P-coated implant	250 mL	NS	7.5 mL	800–1200 × 10^6^/mL	CaCl_2_/ bovine thrombin	NS	P3-x	[82]
Rabbit calvaria	PRP	PRP + BMSCs	10 mL	2	1 mL	NS	NS	NS	Not applicable	[83]
Rabbit calvaria	PRP	PRP + induced/uninduced BMSCs	7 mL	2	NS	NS	CaCl_2_/ thrombin	NS	x	[84]
Mice calvaria	PRP	PRP + BMSCs	2.0 mL	2	NS	NS	10% calcium gluconate	NS	x	[85]
Sheep tibia	PRP	hPRP + ADSCs		2	NS	1.0 × 10^9^/mL	CaCl_2_/ thrombin	NS	P3-x	[86]
Nude mice inguinal groove	PRP	hPRP + hADSCs	400 mL	3	NS	NS	Thrombin/ CaCl_2_	NS	x	[87]
Rat calvaria	PRP	PRP + BCP or MSCs	NS	2	0.5 mL	NS	NS	NS	Not applicable	[88]
Rabbit radius	PRP	PRP + CDHA + BMSC	NS	NS	NS	NS	CaCl_2_/ thrombin	NS	x	[89]
Rabbit radius	PRP	PRP + CDHA + MSC	NS	NS	NS	NS	Thrombin/ CaCl_2_	NS	x	[90]
Rabbit ulna	PRP Gel Membrane	PRP + rBMSCs + nanohydroxypaptite poly (ester urethane)	NS	3	NS	3 × 10^6^ platelets/*µ*L	NS	NS	P4	[91]
Rabbit tibia	PRP	PRP + *β*-TCP + BMC	9 mL	1	NS	NS	Calcium gluconate	NS	x	[92]
Nude mice cranium	PRP	PRP + *β*-TCP + BMAC	27 mL	NS	NS	NS	NS	NS	Not applicable	[93]
Rabbit calvaria	PRPGF	PRGF + Nano-HA granule + MSCs	10 mL	1	1 mL	NS	CaCl_2_	NS	x	[94]
Rabbit radius	PRP	PRP + DPB + MSCs	60 mL	2	NS	12.87 ± 0.848 × 10^6^/*μ*L	CaCl_2_/ thrombin	NS	P4-x	[95]
Rat calvaria	PRP	PRP + BiOss + BMSCs	3 mL	2	0.5 mL	NS	NS	NS	Not applicable	[18]
Rabbit patellar tendon	PRP	PRP	8 mL	1	2 mL	NS	NS	NS	Not applicable	[96]
Rabbit patellar tendon	PRP	PRP	8 mL	1	2 mL	NS	NS	NS	Not applicable	[97]
Rabbit Achilles tendon	PRP	PRP	8 mL	1	2 mL	NS	NS	NS	Not applicable	[98]
Horse SDFT	PRP	PRP	NS	NS	3 mL	NS	NS	NS	Not applicable	[99]
Horse SDFT	PRP	PRP	NS	NS	3 mL	639.7 ± 103.2 × 10^9^/L	NS	42.1 ± 16.7 × 10^9^/L	P2-A	[100]
Rat patellar tendons	PRP	PRP	60 mL	2	NS	523.8 × 10^4^/mL	NS	NS	P1	[101]
Rat rotator cuff	PRP	PRP	5 mL	2	NS	13.8 × 10^9^ platelets/L	NS	NS	P1	[102]
Rat Achilles tendon	PRP	PRP	NS	2	NS	(2.2–2.9) × 10^6^/mm^3^	CaCl_2_	1 × 10^2^/mm^3^	P4-x-B	[103]
Rat tendon-from-bone supraspinatus tear	PRP	PRP	7-8 mL	2	NS	NS	CaCl_2_/thrombin	NS	x	[104]
Rabbit Achilles tendon	PRP	PRP	8 mL	NS	2 mL	NS	NS	NS	Not applicable	[105]
Sheep Achilles tendon	PRGF	PRGF	20 mL	1	2 mL	NS	CaCl_2_	NS	x	[106]
Rabbit intrasynovial flexor tendons	PRPF	PRP/PRPF	10 mL	2	ns	313.5 (±72.3) × 10^4^ *μ*L	CaCl_2_/thrombin	NS	P4-x	[107]
Rat patellar tendon	PC	PC	9 mL	1	0.5 mL	445–862 thousand/mm^3^	NS	NS	P2/P3-x	[108]
Sheep DDFT	PRP	PRP + 10 × 10^6^ autologous PB-MSCs	18 mL	NS	3/5 mL	882 ± 199 × 10^3^ platelet/*μ*L	NS	NS	P3	[109]
Rabbit Achilles tendon	PRP	PRP + 1 × 10^7^ ADSCs	10 mL	2	NS	NS	CaCl_2_	NS	x	[110]
Sheep Achilles tendon	PRPFM	PRPFM + APD	9 mL	NS	NS	NS	NS	NS	Not applicable	[111]
Rat Achilles tendon	PRP	PRP + Collagen sponge + TSCs	20 mL	2	NS	8 × 10^9^ platelets/L	NS	NS	P1	[112]
Mice abdominal cavities	PRCR	10% PRCR in DCs	50 mL	2	NS	10.0 × 10^8^ platelets/mL	CaCl_2_	NS	P3-x	[113]
Rabbit articular cartilage	PRP	PRP + PLGA	16 mL	2	0.8 mL	125.59 × 10^4^/*μ*L	NS	NS	P4	[114]
Sheep articular cartilage	PRP	PRP + collagen-HA	20 mL	1	NS	874 ± 87 × 10^3^/*μ*L	CaCl_2_	NS	P3-x	[115]
Caprine articular cartilage	PRP	PRP + DBM	27 mL	1	3 mL	1511 × 10^9^/L	NS	NS	P4	[116]
Rabbit articular cartilage	PRP	PRP + chondrocytes	10 mL	2	1 mL	NS	NS	NS	Not applicable	[117]
Rabbit articular cartilage	PRP	PRP + BMSCs PRP + ADSCs	18 mL	2	0.5 mL	16.7 ± 1.1 × 10^8^/mL	Ca	NS	P4-x	[118]
Rabbit articular cartilage	PRP	PRP + SDMC	9 mL	2	1 mL	2103.56 ± 479.5 × 10^3^/mL	NS	NS	P4	[119]
Rat articular cartilage	PRP	PRP + hydrogel + chondrocytes	40 mL	2	NS	1240.9 ± 86.1 × 10^4^/*µ*L	NS	NS	P4	[120]
Rabbit articular cartilage	PRP	PRP + hydrogel + chondrocytes	10 mL	2	NS	NS	NS	NS	Not applicable	[121]
Mini pig articular cartilage	PRP	PRP + BMSCs + biphasic scaffold	60 mL	1	10 mL	2.2 ± 0.57 × 10^6^ cells	Thrombin/CaCl_2_	NS	Not applicable	[122]
Rabbit articular cartilage	PRP	Autologous cartilage fragments + hyaluronic acid derivative + human fibrin glue + PRP	8 mL	2	400 *µ*L	858 × 10^3^ platelets/mm^3^ (range 642–1,085)	NS	NS	P3	[123]
Sheep cartilage	PRP	PRP + microfractures	60 mL	2	6–8 mL	1415 ± 164 × 10^3^/mL	Fibrinogen Thrombin	NS	P1-x	[124]
Rat articular cartilage	PRP	PRP + microfractures	NS	2	NS	13.8 × 10^9^ platelets/L	NS	NS	P1	[125]
Porcine ACL	PRP	Collagen-PRP hydrogel	60 mL	2	9 mL	NS	NS	NS	Not applicable	[126]
Canine ACL	PRP	Collagen-PRP hydrogel	NS	1	NS	NS	NS	NS	Not applicable	[127]
Canine ACL	PRP	Collagen-PRP hydrogel	NS	NS	NS	NS	NS	+	Not applicable	[128]
Mini. pig ACL	PRP	3X or 5X PRP + collagen scaffold	NS	NS	NS	1951 ± 304 × 10^9^/L 1161 ± 179 × 10^9^/L	NS	NS	P4 P3	[129]
Rabbit ACL	PRP	PRP + DPB	NS	2	NS	NS	CaCl_2_/bovine thrombin	NS	x	[130]
Canine ACL	PRP	PRP	20 mL	3	1 mL	669 ± 51 × 10^9^ platelets/L	NS	NS	P2	[131]

Not specified (NS), biphasic calcium phosphate (BCP), mesenchymal stem cell (MSC), beta-tricalcium phosphate (*β*-TCP), polytetrafluoroethylene (PTFE), platelet-rich growth factor (PRGF), autologous bone (AB), hydroxyapatite (HA), calcium phosphate cement (CPC), bone marrow mesenchymal stem cells (BMSCs), calcium aluminate (CA), rabbit BMSC (rBMSC), deproteinized bovine bone mineral (DBBM), calcium-deficient hydroxyapatite (CDHA), allogeneic-mesenchymal stem cells (aMSC), titanium (Ti), human adipose derived stem cell (hADSC), human PRP (hPRP), fresh frozen bone allograft (FFBA), autogenous bone (AB), hyperbaric oxygen (HBO) therapy, autologous platelet concentrate (APC), adipose tissue derived stem cells (ASC), calcium phosphate (Ca-P), autogenous particled bone (APB), allogeneic BMSC (aBMSCs), acellular porcine dermal patch (APD) polyglycolic acid (PGA), recombinant human bone morphogenetic proteins (rhBMP2), platelet-poor plasma (PPP), rat PRP (rPRP), human PRP (hPRP), goat (gPRP), perforated bone matrix (PDBM), bone morphogenetic proteins (BMP), polycaprolactone-20% tricalcium phosphate (PCL-TCP), (5-[4-phenyl-5-(trifluoromethyl) thiophen-2-yl]-3-[3-(trifluoromethyl) phenyl] 1,2,4-oxadiazole) SEW2871, borate glass (BG), platelet-rich fibrin (PRF), deproteinized bone matrix (DPB), low-medium-high PRP (L-M-H PRP), bone marrow cells (BMC), adipose derived stem cell (ADSC) bone marrow aspirate concentrate (BMAC), superficial digital flexor tendon (SDFT), deep digital flexor tendon (DDFT), anterior cruciate ligament (ACL), platelet-rich plasma fibrin matrix (PRPFM), concentrate plasma (PC), platelet-rich plasma-clot release (PRCR), plasma rich in growth factors (PRGF), PRP with fibrin matrix (PRPF), tendon stem cells (TSCs), periferal blood mesenchymal stem cells (PB-MSCs), adipose derived mesenchymal stem cells (ADSCs), white blood cells (WBC), poly(lactic-co-glycolic acid) (PLGA), demineralized bone matrix (DBM), hydroxyapatite (HA), synovial membrane derived mesenchymal stem cell (SDMC), bone marrow mesenchymal stem cell (BMSC), adipose derived stem cell (ADSC), deproteinized bone (DPB), and calcium chloride (CaCl2), calcium (Ca).

^*^Anitua method. °Sonnleitner method.

PAW classification: P1 (≤baseline), P2 (>baseline-750,000), P3 (>750,000–1,250,000), P4 (>1,250,000), x (exogenous activation), A (above baseline), B (below baseline), h (human), and g (goat).
